# Single-cell transcriptomics: A new tool for studying diabetic kidney disease

**DOI:** 10.3389/fphys.2022.1053850

**Published:** 2023-01-04

**Authors:** Zi-Hui Mao, Zhong-Xiuzi Gao, Yong Liu, Dong-Wei Liu, Zhang-Suo Liu, Peng Wu

**Affiliations:** ^1^ Traditional Chinese Medicine Integrated Department of Nephrology, The First Affiliated Hospital of Zhengzhou University, Zhengzhou, China; ^2^ Institute of Nephrology, Zhengzhou University, Zhengzhou, China; ^3^ Henan Province Research Center for Kidney Disease, Zhengzhou, China; ^4^ Key Laboratory of Precision Diagnosis and Treatment for Chronic Kidney Disease in Henan Province, Zhengzhou, China

**Keywords:** single-cell RNA-seq, single-nucleus RNA-seq, spatial transcriptomics, diabetic kidney disease, signaling pathway

## Abstract

The kidney is a complex organ comprising various functional partitions and special cell types that play important roles in maintaining homeostasis in the body. Diabetic kidney disease (DKD) is the leading cause of end-stage renal disease and is an independent risk factor for cardiovascular diseases. Owing to the complexity and heterogeneity of kidney structure and function, the mechanism of DKD development has not been fully elucidated. Single-cell sequencing, including transcriptomics, epigenetics, metabolomics, and proteomics etc., is a powerful technology that enables the analysis of specific cell types and states, specifically expressed genes or pathways, cell differentiation trajectories, intercellular communication, and regulation or co-expression of genes in various diseases. Compared with other omics, RNA sequencing is a more developed technique with higher utilization of tissues or samples. This article reviewed the application of single-cell transcriptomics in the field of DKD and highlighted the key signaling pathways in specific tissues or cell types involved in the occurrence and development of DKD. The comprehensive understanding of single-cell transcriptomics through single-cell RNA-seq and single-nucleus RNA-seq will provide us new insights into the pathogenesis and treatment strategy of various diseases including DKD.

## Introduction

The kidney is a highly complex organ that removes waste products from the body, maintains fluid balance, and regulates blood pressure. It comprises a wide range of specialized cell types and functional partitions. Understanding kidney function requires knowledge of the role of each cell type in this complex system. Gene regulatory mechanisms that define cell behavior are very important in nephrology and are key to understanding cellular functions. Traditional methods for renal cell type studies rely heavily on microscopy and fluorescence-activated cell sorting (FACS). These methods provide high spatial resolution with limited markers and cannot fully resolve cell types and states, as well as cell heterogeneity (Wu and Humphreys, 2017). Although single-cell population analysis could obtain the average value of a certain parameter, population studies might only acquire the information of some numerically dominant cell populations from complex tissues. Hence, for the analysis of rare or infinitesimal cell types, the sequencing analysis method at the level of single cell is more necessary.

The single-cell sequencing technology enables comprehensive analysis of cell types and states by combing comprehensive genomics with single-cell resolution (Gawad et al., 2016). Recent advances in single-cell genomics have enhanced the ability of scientists to characterize individual cells. It is no longer necessary to measure the expression of multiple genes by immunofluorescence; instead, the expression of thousands of genes in thousands of single cells can be measured simultaneously (Malone et al., 2018). This technique could improve our ability to conduct high-resolution studies on gene expression profiling patterns and also define the global expression profile of individual cells and the heterogeneity in cell populations (Potter, 2018). Single-cell sequencing can be applied for high-throughput sequencing of single cells and is widely used in human cell mapping and assessing cell heterogeneity, cell development and differentiation, and progression in immunity or disease typing.

## Single-cell sequencing technology

The single-cell sequencing technology is used for sequencing and analyzing the genome, transcriptome, and epigenome of cells. It can reveal the gene structure and expression status of individual cells, thereby reflecting the heterogeneity between cells. Single-cell transcriptomics involves a common workflow including sample preparation, single-cell capture, reverse transcription and transcriptome amplification, library generation, high-throughput sequencing, and data analysis.

A major advantage of single-cell RNA-seq (scRNA-seq) over bulk RNA sequencing is its ability to identify rare cell types and to detect their gene expression (Table 1). Single-cell gene expression profiling can reveal specific cell types and states in tissues; however, it is highly dependent on cell viability. An optimal scRNA-seq workflow requires immediate sample preparation. Isolated nuclei can be used in genomic studies to address this problem. Single-nucleus RNA-seq (snRNA-seq) is an alternative method to assess the cell transcriptome by isolating nuclei. Lake et al. provided a normalized approach and compared nuclear and whole-cell expression data (Lake et al., 2017; Lake et al., 2019). SnRNA-seq might be more widely applied in clinical practice because it is tolerant to sample sources, such as temporal neocortical tissues (Busch et al., 2022), human hippocampus (Su et al., 2022), gut tissue samples (Hung et al., 2022), frozen or hard-to-dissociate tumors (Slyper et al., 2020). One study showed that snRNA-seq performed better on inflammatory fibrotic tissues than scRNA-seq. SnRNA-seq can capture more cell types including glomerular endothelial cells, podocytes, mesangial cells, and juxtaglomerular cells. Both dissociation-induced bias and transcriptional stress response to snRNA-seq are correspondingly reduced, and snRNA-seq is highly compatible with frozen samples (Wu et al., 2019). Even though the scRNA-seq or snRNA-seq provide advantage over bulk RNA seq but still suffer from not providing their transcriptome map with spatial context. The recently developed methods such as GeoMx Assay and CosMx assay from NanoString, RNAscope, MIBI, Visium are coming up with spatially resolved transcriptomics/proteomics data. Combining scRNA-seq or snRNA-seq with higher-resolution spatial transcriptomics (ST) will facilitate spatial localization of kidney diseases of different etiologies, thereby providing new insights into the pathogenesis, and enabling consequent development of potential therapeutic targets (Bell and Denby, 2021).

**TABLE 1:** Strengths and weaknesses of bulk RNA-seq, scRNA-seq and snRNA-seq.

	Bulk RNA-seq	ScRNA-seq	SnRNA-seq
Objective	Measure the average gene expression across sampled cells	Measure the gene expression of individual cells	Measure the gene expression of individual nucleus
	Identify differences between samples	Identify differences between cell types/states	Identify differences between cell types
Strengths	Inexpensive	Cell heterogeneity	Compatible with wide range of tissue and samples
	Relatively simple procedures	Discover new cell types or subpopulations	Avoid dissociable cell type bias
	Standardized bioinformatics tools	Observe genomic and transcriptome differences between individual cells	Prevent transcriptional stress response
	Faster and less complex analysis	Lineage trajectory	Capture more cell types (podocytes, mesangial cell et al.)
		Uncover patterns of co-expression in genes	
Weaknesses	Mask cell heterogeneity	Highly dependent on cell viability	Deletion of cytoplasmic RNA
	Loss detailed information of individual cells	Dissociable cell-type bias	Cellular bias
		Transcriptional stress response	Low sequencing depth

Abbreviations: Bulk RNA-seq, bulk RNA sequencing; scRNA-seq, single-cell RNA sequencing; snRNA-seq, single-nucleus RNA sequencing.

ST is an emerging technology. It is capable of investigating gene expression profiles in histological sections while preserving morphological information regarding transcripts and cell types of origin (Ståhl et al., 2016). Spatially resolved transcriptomics was named the Natural Method of the Year 2020 and is a powerful tool to reveal *in situ* transcriptional expression associated with histopathological features (Marx, 2021). The kidneys are composed of epithelial, endothelial, immune, and stromal cells. These cells are anatomically close to one another. ST enables the investigation of *in situ* expression characteristics on histological images of healthy and diseased kidney samples (Melo Ferreira et al., 2021a). Ferreira et al. applied the 10x Genomics Visium ST platform to study epithelial immune crosstalk in two mouse acute kidney injury models (ischemia/reperfusion injury and cecal ligation and puncture) (Melo Ferreira et al., 2021b). In a mouse model of endotoxemia-associated kidney injury, Janosevic et al. used Visium Spatial Gene Expression integration with scRNA-seq to map transcriptomic changes from early injury to the recovery phase of the disease. Using this method, it is possible to map unique subpopulations of endothelial cells at the spatial resolution (Janosevic et al., 2021).

Using laser capture microdissection, single-cell proteomics can be helpful to understand the perturbation of proteins in different kidney sub-compartments and to uncover heterogeneity in complex glomerular/tubular disease phenotypes (Sigdel et al., 2020). With the aid of mass cytometry, high-dimensional single-cell proteomics can analyze renal tumors heterogeneity. In addition, it is also applied to classify the immune cell population and to investigate the expression of stem cell-related markers in renal tumors (Li et al., 2020). Urinary proteomics and single-cell transcriptomics contribute to detecting proteins and molecular pathways. Furthermore, IL-16 is a potentially treatable target and biomarker in lupus nephritis (Fava et al., 2022). Due to the lack of protein amplification capability, it is still a challenge to apply single-cell proteomics in kidney tissue.

## Single-cell transcriptomics studies in nephrology

**FIGURE 1:**
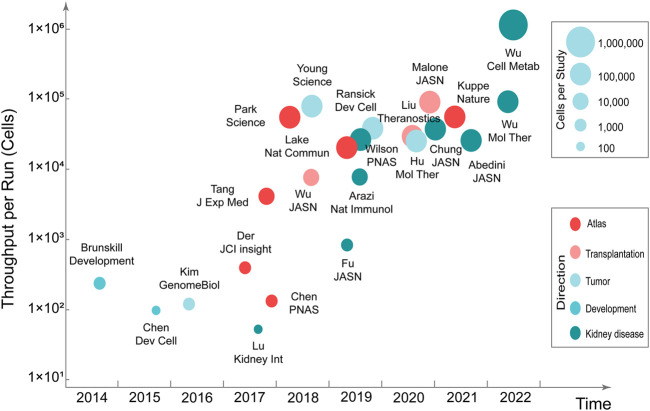
Timeline of single-cell transcriptomics studies in nephrology.

Mapping kidney gene expression is a clear goal of the National Institute of Diabetes and Digestive and Kidney Diseases’ Renal Precision Medicine program. The mapping of kidney gene expression informs about healthy kidney function, and also guides treatment in disease states (kidney-precision-medicine-project-kpmp, 2022). Single-cell transcriptomics can be used to comprehensively characterize human kidney diseases at the cellular level. Compared with single-cell transcriptomics research in the fields of neuroscience, stem cells, and cancer, research in nephrology is still in the developmental stage. Currently, the single-cell transcriptomics has made considerable progress in the research related to kidney development, atlas, renal tumors, disease mechanisms, and therapeutic targets (Stewart and Clatworthy, 2020; Kim and Park, 2021) (Figure 1) (Table 2).

**TABLE 2:** Single-cell transcriptomics studies in nephrology.

Article information	Disease/model	Tissue/cell type	Method (s)	Cell number
Brunskill et al. (2014)	Kidney development	E11.5, E12.5 kidneys, P4 renal vesicles	ScRNA-seq	235
Chen et al. (2015)	Nephron progenitor	Single nephron progenitors	ScRNA-seq & FACS	91
Kim et al. (2016)	Renal cell carcinoma	Primary renal cell carcinoma and its lung metastasis	ScRNA-seq	116
Der et al. (2017)	LN	Human renal and skin biopsy tissues	ScRNA-seq	899
(Lu et al., 2017b; 2017a)	Healthy mouse	glomerular mesangial cells, podocytes	ScRNA-seq	34
Tang et al. (2017)	Adult zebrafish	Zebrafish kidney marrow	ScRNA-seq	11,573
Chen et al. (2017)	Healthy mouse	Renal collecting duct	ScRNA-seq & FACS	218
Park et al. (2018)	Healthy mouse	Kidney	ScRNA-seq	57,979
Wu et al. (2018)	Human kidney allograft	healthy adult kidney	ScRNA-seq	8,746
		Kidney transplant biopsy		
Young et al. (2018)	Renal tumor	Human renal tumors	ScRNA-seq	72,501
		Normal tissue from fetal, pediatric, and adult kidneys		
Fu et al. (2019)	STZ-diabetic eNOS^−/−^ mice	Glomerular cells	ScRNA-seq	829
Lake et al. (2019)	Human adult kidney	Kidney biopsy tissues	SnRNA-seq	17,659
Arazi et al. (2019)	LN	Kidney biopsies, urine, blood	ScRNA-seq	8,961
Wilson et al. (2019)	Early DKD	Kidney	SnRNA-seq	23,980
Ransick et al. (2019)	Adult male and female mouse	Kidney	ScRNA-seq	31,265
Liu et al. (2020)	CKTR	CKTR biopsies	ScRNA-seq	27,197
Hu et al. (2020)	ccRCC	Nephrectomy biopsy samples	ScRNA-seq	24,550
Malone et al. (2020)	Kidney transplants	Human kidney transplant biopsies	ScRNA-seq & Whole-exome sequencing	81,139
Chung et al. (2020)	Nephrotoxic serum nephritis, diabetes, doxorubicin toxicity, CD2AP deficiency	Glomerular cells	ScRNA-seq	75, 000
Kuppe et al. (2021)	CKD	Human kidney epithelial cells	ScRNA-seq & ATAC-seq	53,672
	UUO	Mouse kidney		
Abedini et al. (2021)	DKD	Urine	ScRNA-seq	23,082
Wu et al. (2022c)	Db/db	Kidney	ScRNA-seq	83, 585
Wu et al. (2022b)	Db/db (Lepr−/−)	Kidney	SnRNA-seq & Bulk RNA-seq	946, 660

Abbreviations*:* scRNA-seq, single-cell RNA sequencing; FACS, fluorescence-activated cell sorting; LN, lupus nephritis; STZ, streptozotocin; eNOS, endothelial nitric oxide synthase; snRNA-seq, single-nucleus RNA-seq; DKD, diabetic kidney disease; CKTR, chronic kidney transplant rejection; ccRCC, clear-cell renal cell carcinoma; CKD, chronic kidney disease; UUO, unilateral ureteral obstruction; ATAC-seq, assay for transposase-accessible chromatin using sequencing.

Researchers initially applied single-cell transcriptomics to kidney development studies. Studies revealed early molecular mechanisms of renal precursor patterns and age-dependent gene expression patterns (Brunskill et al., 2014; Chen et al., 2015). Through scRNA-seq, *in situ* expression, and cell lineage tracking of tissues in different parts of the kidneys of male and female mice, it was discovered that the development time and lineage aggregation of kidney units determined cell diversity, significant sex differences, different tissues, and cell composition of kidney units (Ransick et al., 2019). Studies of glomerular mesangial cells have uncovered the heterogeneity and lineage tracking of mesangial cells (Lu et al., 2017b), whereas studies on glomerular podocytes have revealed a list of genes necessary for the cytoskeletons of podocytes (Lu et al., 2017a). Collecting duct cells obtained by FACS enrichment revealed possible intercellular crosstalk (Chen et al., 2017). Single-cell transcriptome analysis of the adult zebrafish kidney and bone marrow cells defined single-cell heterogeneity of zebrafish marrow and kidney cells (Tang et al., 2017). With the development of technology and enhancement of the high-throughput processing of cells, an atlas of adult mouse kidneys has been mapped. The atlas contains 18 previously known cell types and three new cell types (Park et al., 2018). Researchers have analyzed cells throughout their life cycle by mapping healthy adult and fetal kidney immune cells (Stewart et al., 2019). Several major cell types and their molecular characteristics in the kidney were identified through human kidney cell atlas analysis, which provides a strong basis for studying kidney physiology and pathology (Lake et al., 2019).

Studies on kidney disease have found that different models of glomerular injury exhibit different transcriptional responses and associated gene expression patterns (Chung et al., 2020). Clinical parameters (e.g., proteinuria, IgG deposition) were determined by analyzing cells isolated from biopsy specimens of patients with lupus nephritis, which are associated with interferon response genes in renal tubular cells (Der et al., 2017). The pathogenesis of lupus nephritis was discussed in a comparison between patients and healthy controls (Arazi et al., 2019; Der et al., 2019). These studies uncovered 21 subsets of leukocytes active in disease, showing the presence of pro-inflammatory responses and inflammation-resolving responses in lupus nephritis. Moreover, analysis of tubular cells from lupus nephritis patients indicated that inflammation and fibrosis pathways contribute to their histological differences. Kuppe et al. generated a single-cell atlas of the human kidney, focusing on the development of tubular interstitial fibrosis in chronic kidney disease (Kuppe et al., 2021). In renal tumors, researchers mainly explore the cell types, heterogeneity of tumors, and diversity of tumor tissues, providing a reference value for the design of anti-tumor protocols and precision medicine (Kim et al., 2016; Young et al., 2018; Hu et al., 2020).

Studies of the healthy adult kidney and kidney transplant biopsies revealed heterogeneous immune responses in mixed rejection (Wu et al., 2018). By evaluating scRNA-seq profile, chronic kidney transplant rejection is characterized by increased numbers of immune cells and MyoF, resulted in renal rejection and fibrosis (Liu et al., 2020). Donor-origin macrophages and T cells have distinct transcriptional profiles compared with their recipient counterparts. Furthermore, donor leukocytes play a role in rejection (Malone et al., 2020). Overexpressed genes in antibody-mediated rejection were demonstrated in peripheral blood scRNA-seq from kidney transplant patients and kidney allograft biopsies scRNA-seq analysis (Van Loon et al., 2022).

## Application of single-cell transcriptomics in diabetic kidney disease

Diabetic kidney disease (DKD) is a microvascular complication associated with type I and type II diabetes. As an independent risk factor for cardiovascular diseases, DKD has become a public health problem with an increase in prevalence, posing a serious threat to human health and life. DKD is a major cause of end-stage renal disease (ESRD), leading to kidney failure, cardiovascular diseases, and premature death. DKD accounts for more than half of all patients enrolled in renal replacement therapy programs (Thomas et al., 2015). An in-depth understanding of the pathogenesis of DKD is necessary to develop innovative therapeutic strategies to prevent, stop, and reverse DKD. The complexity and heterogeneity of kidney structure and function in healthy and diseased states make it challenging to understand the mechanisms underlying the development and progression of DKD. Therefore, single-cell transcriptomics is ideal for studying specific cell types and states, specifically expressed genes or pathways, cell differentiation trajectories, and regulation or co-expression of genes in DKD (Kaur and Advani, 2021). This chapter reviews the currently published studies on DKD-related single-cell transcriptomics (Table 3).

**TABLE 3:** Application of single-cell transcriptomics in diabetic kidney disease.

Article information	Disease/model	Tissue type	Sample number	Single-cell technique	Cell number
Mouse					
Fu et al. (2019)	STZ-diabetic eNOS^−/−^ mice	Glomerular cells	3 Controls	Fluidigm C1 & Illumina NextSeq 500	829
			3 Diabetic mice		
Chung et al. (2020)	Healthy, nephrotoxic serum nephritis, diabetes, doxorubicin toxicity, CD2AP deficiency	Glomerular cells	3 Controls	10×Genomics Chromium and Illumina HiSeq 4,000	75,000
			4 Nephritis		
			6 Diabetes		
			2 Doxorubicin		
			2 CD2AP		
Wu et al. (2022c)	Db/db	Kidney	2 Db/m	10×Genomics Chromium and Illumina NovaSeq 6,000	83,585
			8 Vehicle controls		
			6 ARBs		
			8 SGLT2i		
			6 ARB + SGLT2i		
Wu et al. (2022b)	Db/db (Lepr^−/−^)	Kidney	70	snRNA-seq & Bulk RNA-seq	946, 660
Human					
Wilson et al. (2019)	Early DKD	Kidney	3 Controls	10×Genomics Chromium & snRNA-seq	23,980
			3 Diabetics		
Menon et al. (2020)	Early DKD	Kidney biopsy	18 Controls	10×Genomics Chromium and Illumina	111,035
	COVID-19	Urine	44 DKD		25,791
			13 COV urine		
Barwinska et al. (2021)	Nephrectomy, Diabetic kidney biopsy	Kidney cortical interstitium	9 Nephrectomies	LMD and sequenced and sn Drop RNA-seq	—
			6 Diabetic kidney biopsy		
Abedini et al. (2021)	DKD	Urine	1 Control urine	10×Genomics Chromium and Illumina HiSeq 4,000	23,082
			17 Patient urine		
Collins et al. (2022)	DKD	Kidney	9 Non-DKD	LMD and sequenced and sn RNA-seq	58,405 (mouse)
	Db/db		18 DKD kidney biopsy		
			3 Background control		
			5 Db/db		

Abbreviations: STZ, streptozotocin; eNOS, endothelial nitric oxide synthase; DKD, diabetic kidney disease; LMD, laser microdissected; ARBs, angiotensin receptor blockers; SGLT2i, sodium-glucose cotransporter 2 inhibitors; COVID-19, coronavirus disease 2019; COV, coronavirus disease 2019 cohort.

### Experimental DKD

A study on streptozotocin-diabetic eNOS^−/−^ mouse glomerular cells confirmed the expression of glomerular cell-specific markers and identified several new potential markers. The predominant immune cell type in diabetic glomeruli is macrophages, with a greater number of macrophages expressing the M1 phenotype in early stages of DKD. Dynamic changes in differential gene expression of endothelial and mesangial cells in diabetic mice are closely related to DKD. In addition, gene expression analysis has shown that individual cells respond differently to diabetic injury (Fu et al., 2019). By characterizing glomerular cells in healthy mice and in four different disease models (nephrotoxic serum nephritis, diabetes, doxorubicin toxicity, and CD2AP deficiency), seven different cell clusters and new markers of mesangial cells were identified in the scRNA-seq of healthy glomeruli. The disease susceptibility gene atlas revealed that *Dach1* and *Vegfa* were relatively specific to podocytes, whereas *Adamts5* and *Pik3r1* were specifically expressed in mesangial cells. Moreover, the Hippo signaling pathway is involved in the podocyte response to injury (Chung et al., 2020). A renal cell transcriptome study divided db/db mice into four treatment groups: vehicle, angiotensin receptor blockers (ARBs), sodium-glucose cotransporter two inhibitors (SGLT2i), or ARBs plus SGLT2i, with db/m as a control. ARBs provided more anti-inflammatory and anti-fibrotic effects, whereas SGLT2i affected mitochondrial function in the proximal tubules (Wu J. et al., 2022). Another one million cell atlases of mouse DKD and their therapeutic response showed heterogeneity of renal cell responses to DKD treatment. Monotherapy and combination therapies target different cells to induce non-overlapping changes. In contrast, combination therapies are more effective in salvaging DKD-associated transcriptional changes. The SGLT2i signature revealed modulation of alternative splicing for this class of drugs (Wu H. et al., 2022).

### Human DKD

Single-cell transcriptomics of human DKD tissues are also being conducted simultaneously. SnRNA-seq was used to analyze the cells of human DKD renal tissue, and it was found that the endothelium, mesangium, proximal convoluted tubules, and distal convoluted tubules all showed strong angiogenic signatures. The potassium secretion in urine of the thick ascending limb, late distal convoluted tubule, and principal cells of the collecting duct was enhanced, and paracellular calcium and magnesium reabsorption was reduced. Cellular properties associated with glomerular and tubular interstitial damage in DKD have been explored (Wilson et al., 2019). ScRNA-seq results from renal biopsies of healthy donors and patients with DKD combined with urine samples of COVID-19 patients showed that ACE2-positive proximal tubular cells were associated with kidney damage due to SARS-CoV-2 infection (Menon et al., 2020). An unrecognized marker KISS1, expressing unchanged cell type markers (NOTCH3, EGFR, and HEG1), and downregulated cell type markers in DKD (MYH11, LUM, and CCDC3) were detected by laser microdissection (LMD) and snRNA-seq on the cortical interstitium (excluding tubules, glomeruli, and vessels) in nephrectomies and diabetic kidney biopsy specimens. The interstitial pathways enriched included G-protein coupled receptor binding, collagen biosynthesis, extracellular matrix organization, and small-molecule catabolism (Barwinska et al., 2021). By performing single-cell sequencing analysis of the urine of patients with DKD, the cells were divided into three groups of 23 clusters. In combination with published human kidney snRNA-seq and bladder scRNA-seq data, the origin of urine cells and their correlation with kidney or bladder cells can be understood. There is an almost one-to-one correlation between urine and kidney cell clusters. Single-cell analysis of urine can provide genetic and drug targets for kidney diseases, allowing the development of new diagnostic and therapeutic approaches (Abedini et al., 2021). A transcriptional network analysis of RNAseq data from LMD of human glomeruli and PT of DKD and reference nephrectomy samples showed enriched pathways, including rhodopsin-like receptors, olfactory signaling, and ribosomes (protein translation) in PT of human DKD biopsy samples. The translation pathway was also enriched in the glomerulus. The top overlapping pathway found in mouse snRNA-seq PT clusters and human LMD proximal tubular chambers is carboxylic acid catabolism. Using ultra-performance liquid chromatography-mass spectrometry, the fatty acid catabolic pathway was found to be dysregulated in the db/db mouse model. The acetyl-CoA metabolite was downregulated in db/db mice, consistent with the differential expression of the genes *Acoxl* and *Acacb*. These findings suggest that PT alterations and carboxylic acid catabolism during protein translation are key features of human and mouse DKD (Collins et al., 2022).

## Specific cell types and associated signaling pathways for DKD

**FIGURE 2:**
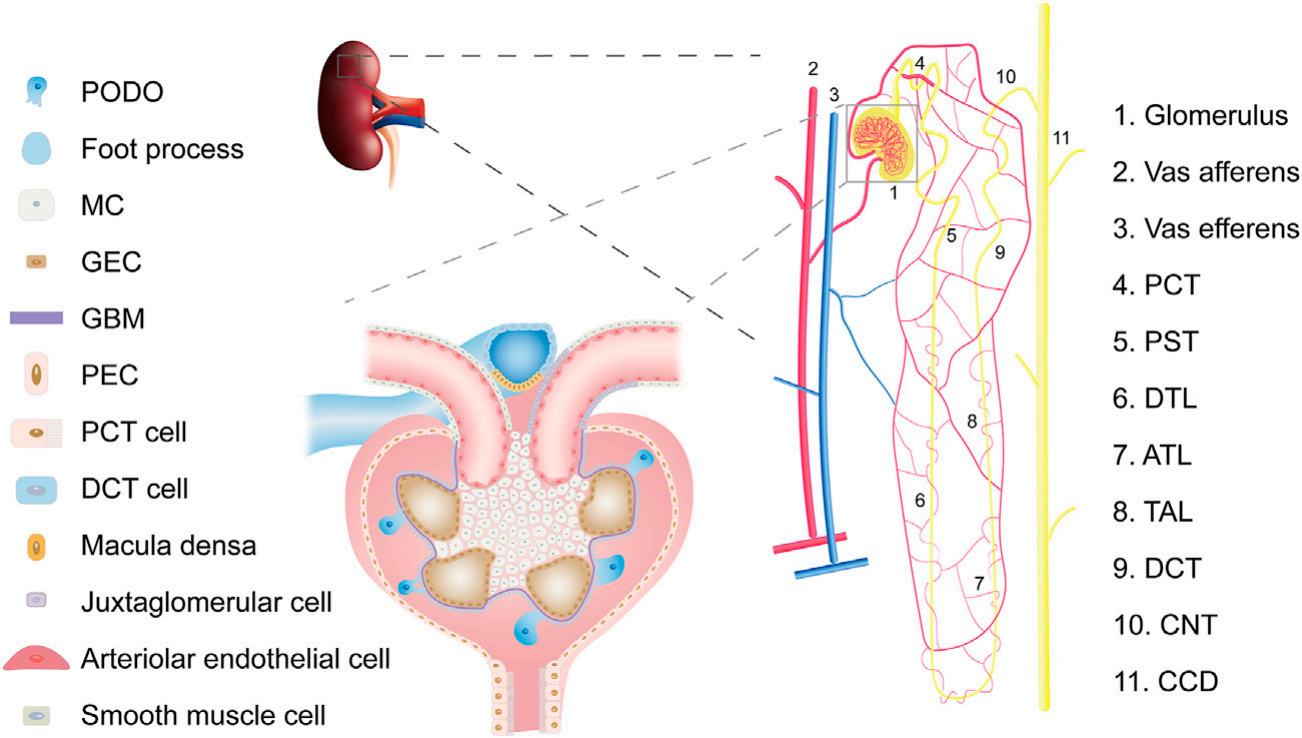
Overview of kidney cell types. Abbreviations: PCT, proximal convoluted tubule; PST, Proximal straight tubule; DTL, descending thin limb of loop of Henle; ATL, ascending thin limb of loop of Henle; TAL, thick ascending limb of loop of Henle; DCT, distal convoluted tubule; CNT, connecting tubule; CD, collecting duct; PODO, podocyte; MC, mesangial cell; GEC, glomerular endothelial cell; GBM, glomerular basement membrane; PEC, parietal epithelial cell.

For a long time, kidney cell types tended to be defined by structure and function (Figure 2). Such a classification ignores the developmental origin and evolution of cells (Park et al., 2019) and fails to elucidate the specific cell subtypes and intercellular heterogeneity. The development of the single-cell sequencing technology has made it possible to achieve strict requirements for unbiased cell type classification (Balzer et al., 2022). It then explores genome-wide gene expression, functional regulation, cell trajectories, and potential crosstalk between cells to improve our understanding of the molecular mechanisms and key drivers of kidney diseases (Humphreys, 2018).

### Glomerulus

The glomerulus is the main unit for renal filtration. Mesangial cells (MCs) maintain their structure, whereas the filtration barrier comprises glomerular endothelial cells (GECs), podocytes (PODOs), and glomerular basement membrane (Karaiskos et al., 2018). Parietal epithelial cell s are unique epithelial cells that surround the Bowman space, and a mouse scRNA-seq study has depicted their global transcriptional profiles (He et al., 2021). The number of GECs in the kidneys of diabetic mice increases, and the number of PODOs and MCs decreases (Fu et al., 2019), which are consistent with angiogenesis disorders and podocyte loss under diabetic conditions (Ziyadeh and Wolf, 2008; Fu et al., 2018). Data on the potential intercellular crosstalk in DKD proposed by scRNA-seq research are relatively limited. Ligand-receptor pairs such as PODOs Vegfa-GECs Flt1 and Kdr, MCs Epha3-GECs Effna1, PODOs BMP7-MCs BMPR2, and other ligand-receptor pairs have been identified in murine glomerular cells (Fu et al., 2019). The expression of CCN1 and SLIT3 in human diabetic MCs was upregulated. The extracellular responding proteins of CCN1, ITGAV and ITGB3/5, are expressed by PODOs and GECs. ROBO2 is expressed by PODOs and GECs that interact with SLIT3 to regulate cell migration (Wilson et al., 2019).

**FIGURE 3:**
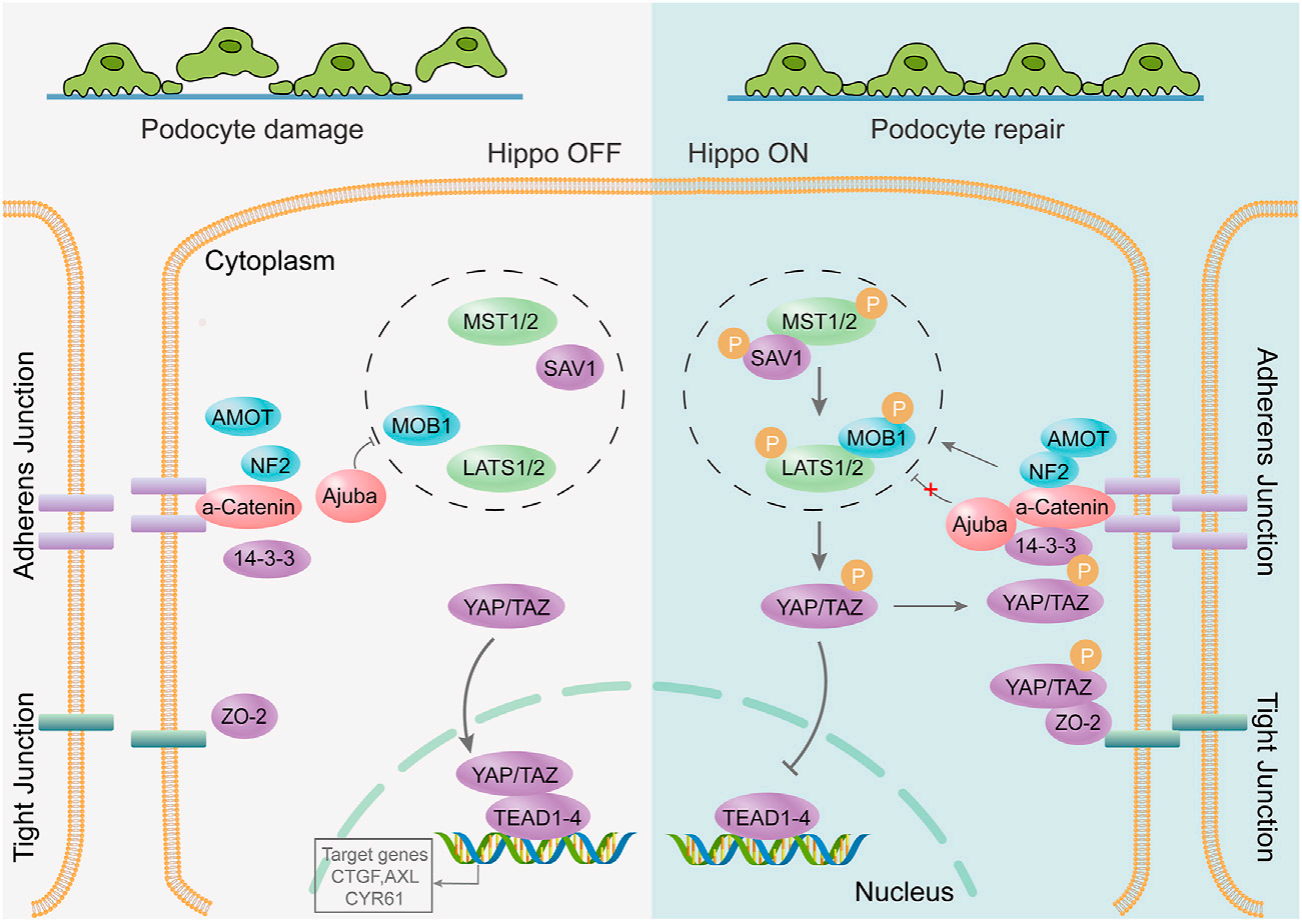
The Hippo signaling pathway is involved in repair after podocyte damage. The podocyte junction is destroyed, the foot process disappears, and the Hippo signaling pathway is activated. YAP/TAZ phosphorylation occurs through a series of cascading phosphorylation reactions. Phosphorylated YAP/TAZ cannot be nucleated to bind to transcription factors; however, it remains within the cytoplasm to function or undergo degradation. It protects podocytes from further damage, thereby promoting post-injury repair.

PODOs are key components of the glomerular filtration barrier; therefore, their damage is an early event in the development of DKD (Ito et al., 2017; Fu et al., 2020). Single-cell transcriptome analysis of glomeruli in healthy mice and four different disease models suggested that the Hippo signaling pathway is critical for repair after podocyte immune damage (Chung et al., 2020). The Hippo signaling pathway regulates biological processes such as cell proliferation, survival, differentiation, determination of cell fate and organ size, and tissue homeostasis (Meng et al., 2016). It plays an important role in cancer occurrence, tissue regeneration, and regulation of stem cell function (Zhao et al., 2011). The key to the Hippo pathway is the complex cascade phosphorylation reaction of serine/threonine-protein kinases. MST1/2 kinases form a complex with SAV1 that phosphorylates and activates LATS1/2. Activated LATS1/2 bind to MOB1A/B, phosphorylates YAP and TAZ (important downstream effectors of the Hippo signaling pathway), and inhibits the transcriptional activity of YAP and TAZ. Conversely, unphosphorylated YAP/TAZ enter the nucleus to bind to TEAD1-4 or other transcription factors, thereby regulating proliferation and downregulating apoptosis-related gene expression (Figure 3). Recent research suggests that the renal protective effects of metformin are also associated with the activation of the Hippo pathway (Corremans et al., 2022). MST1 downregulation promotes renal dysfunction and fibrosis in db/db mice. MST1 overexpression improves renal fibrosis in DKD. Activation of MST1 could be a potential therapeutic strategy for treating or preventing the progression of fibrosis in DKD (Yang et al., 2020). Activation of the Hippo pathway can also inhibit mesangial cell proliferation induced by high glucose levels (Lei et al., 2019), significantly reducing tubular interstitial fibrosis in diabetes (Song et al., 2019; Chen et al., 2020).

### Proximal tubule

Proximal tubule (PT) reabsorbs more than 70% of the original urine through unregulated active and passive paracellular transport (Reidy et al., 2014). According to the differences in morphology, structure, and location, it is divided into three segments (S1, S2, and S3) (Maunsbach, 1966). This classification lacks a discrete transition between morphologies and makes it impossible to clearly cluster cell types that differ from the three groups of cells. Another classification method is to divide PT into proximal convoluted tubule (PCT) and proximal straight tubule (PST), based on the shapes of the tubules and cortex position. The PCT includes S1 (early PCT) and S2 (late PCT), whereas the PST includes S2 (cortical PST) and S3 (medullary PST) (Chen et al., 2019). Targeting PTs with SGLT2 inhibitors can delay DKD progression, further demonstrating the important role of PTs in the prognosis and progression of DKD (Perkovic et al., 2019). A study on human and mouse DKD found carboxylic acid catabolic process, steroid hormone biosynthesis, glutathione metabolism, and biological oxidation were enriched in PT cells. Among these, the carboxylic acid catabolic process is the top pathway identified in mouse snRNA-seq and human datasets (Collins et al., 2022). Spatially resolved metabolomics by mass spectrometry imaging revealed that increased glycolysis, mitochondrial dysfunction, AMPK inhibition, and lipid metabolism disorders occurred in a region-specific manner in the kidneys of rats with DKD (Wang et al., 2021). However, this region-specificity involved only a cortical medullary partition and could not be localized to a particular cell type. SnRNA-seq of fibrotic inflamed kidneys revealed two novel PT populations and previous unidentified tubulointerstitial signaling pathways. Moreover, one cluster of PT cells expressed genes related to proliferation and injury whereas another cluster of PT cells displayed dedifferentiation and pro-inflammatory responses (Wu et al., 2019). Another study of renal injury snRNA-seq also identified a unique PT injury/repair cluster that expressed both the injury marker KIM-1 and the proliferation marker Ki-67 as well as induced the expression of pro-inflammatory cytokines and pro-fibrosis markers (Ma Z. et al., 2022).

**FIGURE 4:**
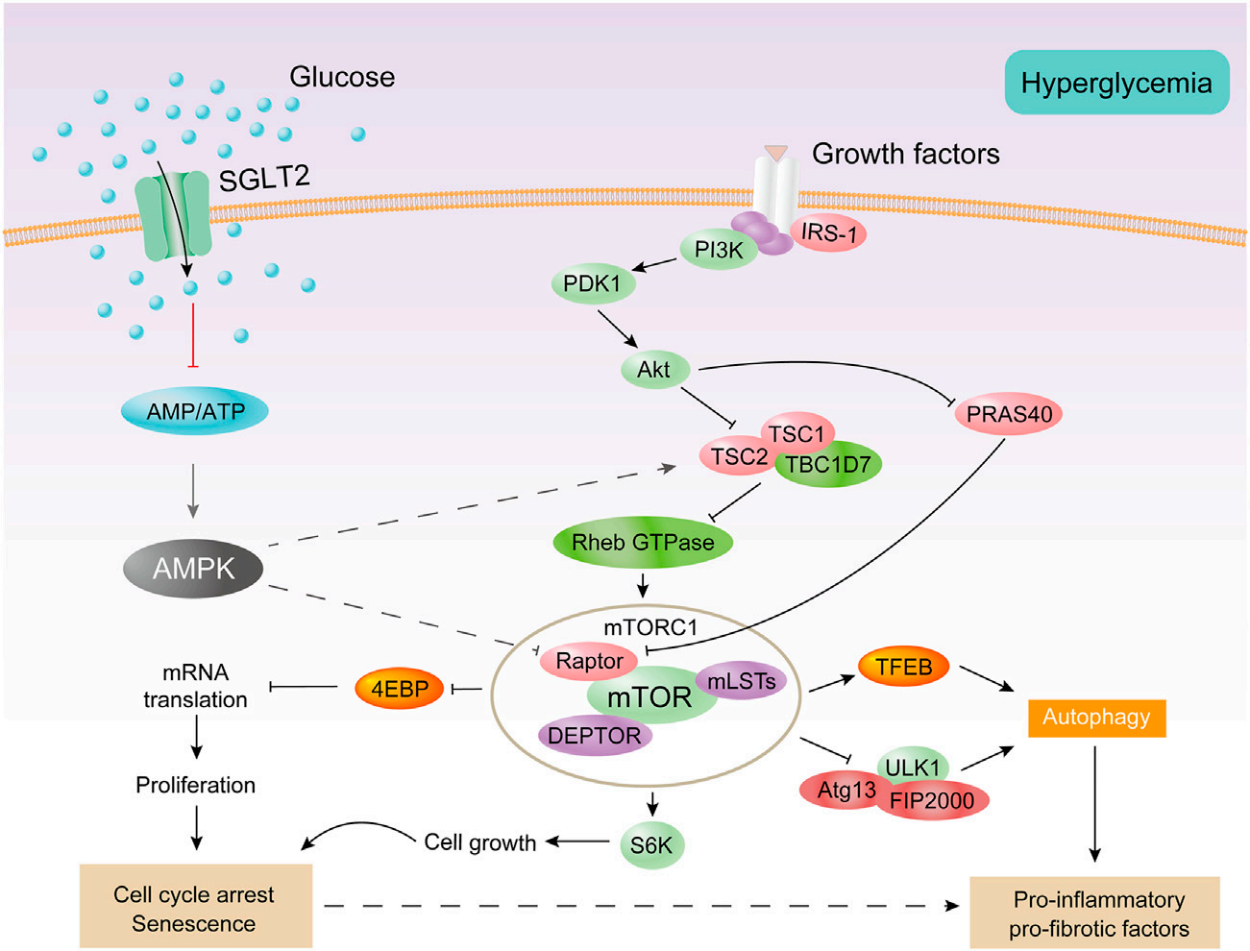
AMPK and mTORC1 are involved in the proximal tubular cell response to diabetes. Under diabetic conditions, excessive energy substrates inhibit AMPK. Filtering and locally producing growth factors as well as hyperglycemia can activate mTORC1. Activation of mTORC1 impairs autophagy and promotes cell proliferation, followed by cell cycle arrest and hypertrophy, thereby producing senescent-like cell phenotypes. All of these processes are associated with inflammation and fibrosis.

In the early stages of diabetes, proximal tubular epithelial cells proliferate in response to a variety of stimuli, including AMPK inhibition and mTORC1 of activation (Vallon and Thomson, 2020). Then, the cell cycle stagnates, hypertrophy occurs, and finally cell senescence is observed (Verzola et al., 2008; Satriano et al., 2010). AMPK is the main regulator of glycolipid metabolism and plays a key role in homeostatic regulation of cellular energy (Zhang et al., 2009; Herzig and Shaw, 2018; Lin and Hardie, 2018). AMPK is a heterotrimer composed of α-catalytic subunit and β-γ regulatory subunits (Hardie et al., 2012). AMPK activation inhibits the activation of mTORC1 (Herzig and Shaw, 2018; González et al., 2020). mTORC1 is a major growth regulatory molecule that consist of mTOR, Raptor, mLST8, and two inhibitory subunits (PRAS40 and DEPTOR). mTORC1 promotes protein and lipid synthesis by phosphorylation of S6K and 4E-BP after sensing and combining different nutritional and environmental factors (such as growth factors, energy levels, cellular stress, and amino acids), and limits autophagy by phosphorylation of ULK1 and ATG13 (Laplante and Sabatini, 2013) (Figure 4). Under diabetic conditions, elevated levels of glucose, amino acids, and insulin activate mTORC1 (Zoncu et al., 2011; Luo et al., 2022). The overactivation of mTORC1 can lead to endoplasmic reticulum stress, oxidative stress, and insulin resistance (Yasuda-Yamahara et al., 2021) and can also inhibit cytoprotective autophagy, leading to worsening of kidney damage in DKD (Kume et al., 2012). mTORC2, another mTOR complex, is also involved in the insulin signaling pathway, which consists of mTOR, Rictor, mLST8, Protor-1, Deptor, and mS1N1. mTORC2 promotes cell survival by phosphorylation of Akt, modulation of the cytoskeleton by activation of PKC and Rho, and phosphorylation of SGK1 to control ion transport (Saxton and Sabatini, 2017). The endoplasmic reticulum stress-related protein TRB3 can reduce proteinuria and inflammatory gene expression in DKD by inhibiting the mTORC2/Akt pathway (Borsting et al., 2014).

### Loop of henle and distal tubule

The loop of Henle consists of six distinct segments: the descending thin limb (DTL) (DTL1, DTL2, and DTL3) (Pannabecker, 2012), ascending thin limb, and thick ascending limb (TAL) (medullary TAL and cortical TAL) (Mount, 2014; Chen et al., 2019). The loop of Henle reabsorbs water and electrolytes and plays a key role in maintaining extracellular fluid volume, urine concentration mechanisms, and urinary protein composition. At the junction of the TAL and distal convoluted tubule (DCT), there is a tightly packed group of specialized cells called macula densa cells. Macular cells are sensor elements of the kidney that detect changes in the composition of distal renal tubular fluid and transmit signals to glomerular vascular elements (Bell et al., 2003). This tubuloglomerular feedback mechanism plays an important role in regulating the glomerular filtration rate and renin production (Briggs and Schnermann, 1987; Kurtz, 2011).

The distal tubule, which is the main site of ion transport and regulation, is located in the middle of the macular densa and the collecting duct. The distal tubule is important in maintaining the acid-base balance of body fluids (Reilly and Ellison, 2000). The distal tubule consists of a DCT and connecting tubule. DCT can be further divided into DCT1 (early DCT) and DCT2 (late DCT), based on different reactions to mineralocorticoid aldosterone. DCT plays a key role in the homeostasis of sodium, potassium, calcium, and magnesium ions (Ar and Dh, 2014). Currently, there are few single-cell studies on DCT and TAL, which may be related to the difficulty of isolation and the small number of obtained single cells. Single-cell transcriptome studies of early human DKD revealed changes in diabetes-induced gene expression, with increased urinary potassium secretion and decreased calcium and magnesium reabsorption (Wilson et al., 2019). The latest single-cell study defined the rare cell types of macular densa cells and renin + cells from the juxtaglomerular apparatus (Wu H. et al., 2022). ScRNA-seq of renal medullary carcinoma revealed a heterogeneous malignant cell lineage originating from the thickened ascending limb of Henle’s loop. This cell exhibited increased resistance to ferroptosis and proteotoxic stress driven by MYC-induced proliferative signaling (Tourigny et al., 2022). SnRNA-seq results from patients with renal injury revealed that the distal convoluted tubules were enriched for novel damage-associated cellular states that drive renal injury through transcriptomic patterns related to oxidative stress, hypoxia, interferon response, and epithelial-mesenchymal transition (Hinze et al., 2022).

### Collecting duct

**FIGURE 5:**
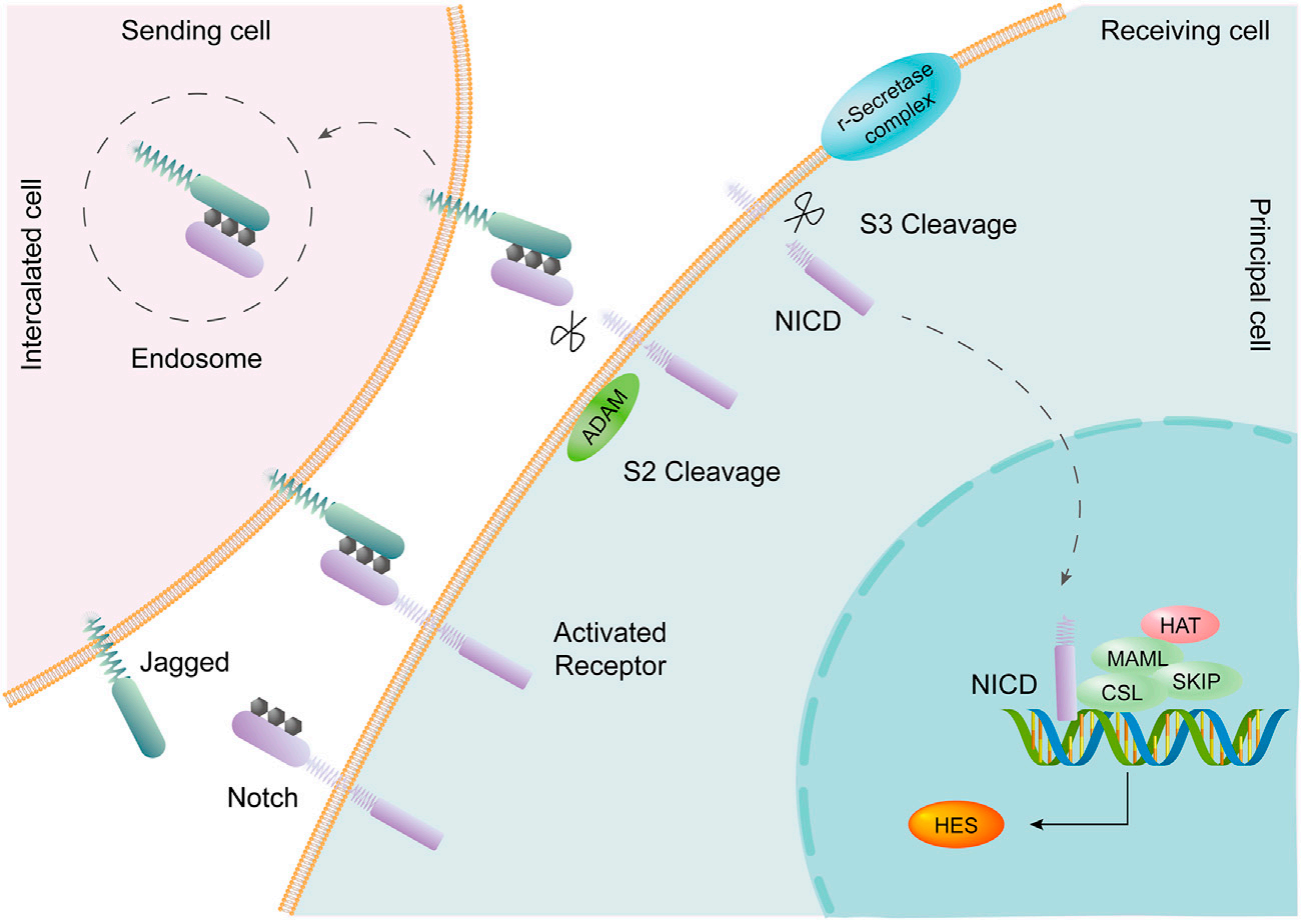
The Notch signaling pathway is involved in transformation between PCs and ICs. Intercalated cells (ICs) express the Notch ligand Jagged. Principal cells (PCs) express the Notch receptor. Activation of the Notch signaling pathway promotes the transition of ICs to PCs in the collecting duct of the adult kidney.

The collecting duct is the final segment of the renal tubule, develops from a ureteral bud, and contains 2 cell types: principal cells (PCs) and intercalated cells (ICs). Single-cell transcriptomes from collecting duct-specific knockout of the transcription factor grainyhead-like two mice showed reduced renal medullary osmolality (Hinze et al., 2021). This technique is helpful for the identification of collecting duct renal cell carcinoma and the exploration of the mechanism of drug resistance, differentiation, and metastasis (Pan et al., 2020). A transitional cell type (PC/IC cell) in which ICs are transformed into PCs was identified through cell trajectory analysis; Notch signal transduction plays an important role in this process (Mukherjee et al., 2019). PCs express the Notch receptor, and ICs express the Notch ligand (Chen et al., 2017). The change in cell fate from ICs to PCs may be the cause of metabolic acidosis (Park et al., 2018). The Notch signaling pathway is an important pathway that affects cell fate, including cell differentiation, proliferation, and apoptosis, and boundary formation, through communication between adjacent cells (Andersson and Lendahl, 2014). In mammals, there are four Notch receptors (Notch1-Notch4) and five Notch ligands (Delta1, Delta3, Delta4, Jagged1, and Jagged2), all of which are transmembrane proteins. The Notch signal is generated by binding to the receptor by the adjacent cell Notch ligand, and the Notch receptor undergoes proteolytic digestion to form a soluble NICD (activated form of the Notch protein), which is transferred to the nucleus to bind to the CSL protein, activates the transcription of the target gene, and plays a biological function (Strooper et al., 1999; Sirin and Susztak, 2012) (Figure 5).

In DKD, the Notch signaling pathway is significantly activated, mediating glomerular function, podocyte damage, and tubular interstitial fibrosis (Zhang et al., 2021; Li et al., 2022). A randomized controlled clinical study showed that elevated Notch2 and Jagged1 gene expression levels were associated with DKD in humans and could serve as potential biomarkers for DKD (Al-Awaida et al., 2021). In a study of renal biopsies in patients with DKD and animal models of DKD, it was found that interaction of the Notch signaling pathway with the NF-κB signaling pathway in macrophages promoted polarization of the macrophages and exacerbated the inflammatory response, fibrosis, and necrosis of the intrinsic cells in the kidneys. Inhibition of the Notch signaling pathway in macrophages can alleviate renal pathological changes (Ma T. et al., 2022). Microencapsulated islet transplantation mitigates PODO damage caused by DKD through inhibiting the Notch signaling pathway (Yuan et al., 2022).

### Immune cells

Inflammation is also involved in DKD pathogenesis and progression. The Activation of innate immune cells plays a key role in triggering and maintaining inflammation (Tang and Yiu, 2020). The increase in the number of immune cells in diabetic glomeruli is consistent with the involvement of inflammation in the occurrence and development of DKD (Navarro-González et al., 2011), particularly macrophages (M1 phenotype-dominant), indicating that macrophages are the main immune cells in diabetic glomeruli and can lead to diabetic kidney damage. The number of macrophages in diabetic glomeruli is closely related to the clinical outcome and prognosis in DKD (You et al., 2013; Klessens et al., 2017). TNFRSF21 is a urine marker of the kidney risk inflammatory signature associated with ESRD. The upregulation of TNFRSF21 expression in infiltrated monocytes in diabetic samples suggests that monocytes also play an important role in the pathogenesis of DKD (Wilson et al., 2019). High-dimensional cellular analyses such as single-cell RNA sequencing applied to patients’ renal-biopsy samples (even frozen samples) can determine the phenotype of infiltrating and resident immune cells as well as parenchymal cells in the kidney (Rao et al., 2020). An integrated scRNA-seq, bulk RNA-seq, and mass spectrometry-based proteomics research revealed that inflammation was induced by activation and recruitment of cytotoxic T cells and macrophage M1 cells is a major contributor to permanent kidney injury (Chen et al., 2022). An enormous scRNA-seq profile of immune cells generated in mouse model of DKD highlights the pivotal role of leukocyte subtypes in DKD (Wu and Humphreys, 2022).

## Limitations of single-cell transcriptomics in DKD

The single-cell sequencing technology captures individual cells, obtains expression information from individual cells, and detects heterogeneity data of cells in promiscuous samples. Bulk RNA-seq combined with microdissection can characterize transcripts and pathways in glomeruli and tubules (Levin et al., 2020). However, the results of single-cell sequencing are more likely to reflect differences in gene expression at the cellular level, observing genomic and transcriptome differences between individual cells. Circulating peripheral blood mononuclear cells (PBMCs) can be utilized to check immunoreaction and inflammatory injury, and to acquire valuable markers associated with diabetic kidney disease (Hodgkinson et al., 2001; Huang et al., 2022). However, in comparison with cells isolated from kidney tissue by scRNA-seq, PBMCs is not sufficient to describe kidney innate cells (podocyte, mesangial cell, tubular epithelial cell, and renal interstitial fibroblasts et al.) since it usually just focuses on lymphocyte and monocyte. Although the single-cell transcriptomics has achieved considerable advance in kidney physiology and disease research, several challenges remain in human kidney research. Human kidney specimens are difficult to obtain, and the sample size is usually small. The availability of human kidney tissues is difficult to predict, and tissue storage protocols still require optimization. Adults have a relatively dense renal matrix and exhibit difficulty in enzymatic dissociation. Enzymatic protocols often impair cell viability, and producing high-quality single-cell suspensions is challenging. SnRNA-seq has more advantages than scRNA-seq; however, it leads to deletion of mitochondrial RNA. Although kidney tissues in mouse DKD models are readily available, there are differences between species and their specific responses to treatment.

Although the single-cell sequencing technology has developed rapidly in recent years, the cost of this technology remains relatively high. As the ability to process cells with high throughput increases, interpreting sequencing experimental data become a major challenge. Another problem is the use of different function parameters and other variables in the bioinformatic processing of single-cell experimental data. When evaluating sequencing data, careful attention must be paid to biological fitness (Malone, 2021). For low- or moderately expressed transcripts, “transcript loss” occurs; therefore, despite the continuous expression of a particular protein, its transcript may not be detectable for a considerable period of time. Proteomic analysis provides the advantage of analyzing protein levels that are closely related to function (Park et al., 2019). The use of different sequencing platforms and library preparation methods makes it relatively difficult to make valuable comparisons between studies.

## Summaries and perspectives

In terms of clinical applications, cell type-specific gene expression associated with chronic kidney disease, diabetes, and hypertension can help identify disease-specific targets, thereby guiding the development of targeted drug therapy (Lake et al., 2019). Based on molecular data from pathogenic mechanism studies and the discovery of potential biomarkers, the diagnosis and treatment of various kidney diseases can be improved (Malone et al., 2018). Because multiple kidney cells can be sequenced from urine, there are alternative options to invasive procedures and difficult-to-obtain samples, and sequencing is becoming more patient-oriented (Wu H. H. L. et al., 2022). Combining single-cell genomics, emerging ST, proteomics, multiplex phenotyping, and functional analysis helps develop next-generation diagnostic and therapeutic approaches (Hansen et al., 2022; Kuppe et al., 2022). A comprehensive understanding of the different cell profiles of kidney types and the signaling pathways in these cell types provides an extremely valuable resource for understanding the cell origin and functional heterogeneity of different kidney diseases from multiple perspectives. With the continuous development of the single-cell sequencing technology, more benefical results will be achieved in the fields of medicine and healthcare, and it will become an indispensable technology for the treatment of diseases and exploration in life sciences.

This review summarizes the application of the single-cell transcriptomics in nephrology, particularly regarding DKD. Although there are still some shortcomings and challenges, with its development, the single-cell sequencing technology has broad application prospects in areas such as kidney health, disease mechanism research, and drug treatment targets.
